# Next-generation genome sequencing can be used to rapidly characterise sequences flanking T-DNA insertions in random insertional mutants of *Leptosphaeria maculans*

**DOI:** 10.1186/s40694-014-0010-y

**Published:** 2014-12-07

**Authors:** Kylie Chambers, Rohan GT Lowe, Barbara J Howlett, Manuel Zander, Jacqueline Batley, Angela P Van de Wouw, Candace E Elliott

**Affiliations:** 1 School of Botany, the University of Melbourne, Parkville, 3010 Victoria Australia; 2 Department of Biochemistry, La Trobe University, Bundoora, 3086 Victoria Australia; 3 School of Agriculture and Food Sciences, University of Queensland, St Lucia, Brisbane, 4072 Queensland Australia; 4 School of Plant Biology, University of Western Australia, Crawley, 6009 Western Australia Australia

**Keywords:** Next-generation sequencing, Filamentous fungi, T-DNA mutants, TAIL-PCR

## Abstract

**Background:**

Banks of mutants with random insertions of T-DNA from *Agrobacterium tumefaciens* are often used in forward genetics approaches to identify phenotypes of interest. Upon identification of mutants of interest, the flanking sequences of the inserted T-DNA must be identified so that the mutated gene can be characterised. However, for many fungi, this task is not trivial as widely used PCR-based methods such as thermal asymmetric interlaced polymerase chain reaction (TAIL-PCR) are not successful.

**Findings:**

Next-generation Illumina sequencing was used to locate T-DNA insertion sites in four mutants of *Leptosphaeria maculans*, a fungal plant pathogen. Sequence reads of up to 150 bp and coverage ranging from 6 to 24 times, were sufficient for identification of insertion sites in all mutants. All T-DNA border sequences were truncated to different extents. Additionally, next-generation sequencing revealed chromosomal rearrangements associated with the insertion in one of the mutants.

**Conclusions:**

Next-generation sequencing is a cost-effective and rapid method of identifying sites of T-DNA insertions, and associated genomic rearrangements in *Leptosphaeria maculans* and potentially in other fungal species.

## Findings

Forward genetics approaches, where banks of mutants with random insertions of DNA are screened for phenotype changes, are often used to study fungi that are recalcitrant to reverse genetics approaches such as gene deletion by homologous recombination [1]-[6]. The inserted DNA, often T-DNA from *Agrobacterium tumefaciens*, usually integrates at a single site [4]. Multiple isolates with random insertions are generated and screened for the phenotype of interest. The insertion site is then identified by thermal asymmetric interlaced polymerase chain reaction (TAIL-PCR) or plasmid rescue [1],[4]. For TAIL-PCR, sequence flanking the T-DNA insertion is amplified using a set of nested T-DNA-specific primers paired with a short degenerate primer. TAIL-PCR has been used to obtain 74 to 100% of flanking sequences in fungi such as *Fusarium oxysporum*, *Magnaporthe oryzae*, *Histoplasma capsulatum*, as well as the lichen fungus *Umbilicaria muehlenbergii* [2],[7]-[9]. However, the rate of success in identifying insertions in other fungi such as *Trichoderma reesei*, and the ascomycete plant pathogens *Colletotrichum higginsianum* and *Leptosphaeria maculans*, ranges from 30 to 66% [1],[10],[11]. For example; only 135 out of 400 (33.7%) flanking sequences were retrieved from the right borders in an analysis of T-DNA insertional mutants of the *L. maculans* [1]. In plasmid rescue, genomic DNA of the T-DNA-containing mutant is cut with a restriction enzyme and then circularized with T4 DNA ligase, thus releasing a fragment of the T-DNA, a selectable marker and flanking genomic sequences [3],[4]. This method requires the use of a T-DNA plasmid containing a bacterial origin of replication and selectable marker and its successful use depends upon the proximity of appropriate restriction enzyme sites to the T-DNA insertion. The use of next-generation sequencing, for identifying the T-DNA insertion site, can overcome these limitations. In this paper we report the use of Illumina sequencing to identify T-DNA flanking sequences in four loss-of-pathogenicity mutants identified from a bank of *L. maculans* insertional mutants.

*L. maculans* isolate IBCN18 was transformed with plasmids pKHT or pKO21 [4],[12]. Plasmid pKO21 is a modified version of pPZPtk8.10, which has a hygromycin resistance gene regulated by the *A. nidulans* trpC promoter and terminator sequences inserted into the multiple cloning site. Four insertional mutants, UM176, UM314, UM316, UM317, were identified with a loss-of-pathogenicity phenotype on cotyledons of *Brassica napus*, as described previously [13]. Genomic DNA (4 μg) was prepared from mycelia [14], digested with restriction enzymes, electrophoresed and then probed with the hygromycin resistance gene on a Southern analysis blot (data not shown). This confirmed that UM176, UM316 and UM317 each carried a single T-DNA whereas UM314 had two independent insertions of T-DNA.

Identification of the corresponding insertion sites in the four mutants was attempted using TAIL-PCR. Six different degenerate primers were used in combination with border-specific primers to amplify either the left border (LB) or right border (RB) sequences [4],[15]. No amplicons containing the corresponding T-DNA sequence were produced using any combination of TAIL-PCR primers. Plasmid rescue failed to retrieve flanking sequence from UM176, in which the T-DNA was derived from pKHT. Since both TAIL-PCR and plasmid rescue failed to identify insertion sites, Illumina next-generation sequencing was then undertaken.

Genomic DNA was extracted from mycelia, then treated with RNaseA (Invitrogen). Sequencing libraries (NexteraXT; Illumina) were prepared, pooled and run on a MiSeq desktop sequencer (Illumina) [16]. Paired-end reads (150 bp) were generated, with genome coverage for the four mutants ranging from 6.6×-24× (Table 1). Sequencing reads were filtered to remove those of low quality and/or high percentage of Ns; adapter sequences were also removed. Separate BLASTN databases were generated for the sequence reads of each of the mutants using Geneious R6.0.5 (BLAST version Blast 2.2.28+). These BLAST databases were then queried using the sequences of the region between the LB and RB of the T-DNA insert. All reads that matched the T-DNA query, with expect scores of 1e^−10^, were retrieved and aligned to the query sequence. Flanking sequences that extended beyond the T-DNA query sequence were then used for sequence walking by querying the BLAST database again and thus retrieving more flanking sequences. This process of sequence walking was continued until enough flanking sequence was obtained to BLAST the GenBank nr database to identify neighboring regions in the *L. maculans* genome. Based on previous studies, a minimum alignment length of 15 bp unambiguously identifies read locations [17]. Thus to identify an insertion site, a read needs to contain at least 15 bp of the T-DNA sequence, as well as at least 15 bp of *L. maculans* genome sequence. Therefore for each 150 bp read, there are 120 consecutive binding sites that meet these criteria. The genome of *L. maculans* is approximately 45 Mbp [18]. If each read can bind to 120 consecutive loci in the genome then the probability of identifying a T-DNA flanking sequence is 2.6 × E^−06^ (120 bp/45,000,000 bp) or 1 in 345,000 reads. The predicted number of reads that should flank the T-DNA insertion site for each mutant, based on these criteria, is very similar to the actual number of reads identified (Table 1). A T-DNA insertion site was located in each of the four mutants (Table 1).

**Table 1:** **Features of flanking regions of insertional mutants of** ***Leptosphaeria maculans*** **analysed by Illumina next**-**generation sequencing**

Mutant	Plasmid inserted	Number of reads (Genome coverage)	Number of reads flanking each border (LB, RB)		Number of nucleotides deleted from T- DNA borders (LB, RB)	Number of nucleotides deleted from ***L. maculans*** DNA (bp)	Genes adjacent to T- DNA insertion site
			Theoretical	Observed			
UM176	pKHT	4361600 (14.5×)	11.6, 11.6	16, 12	261 , 4	26,312	Between Lema_G56010 and Lema_G101830^b^
UM314^a^	pKO21	7219200 (24×)	19.3, 19.3	12, 11	43, 44	16	Within Lema_G051670
UM316	pKO21	1985280 (6.6×)	5.3, 5.3	4, 0	87, RB deleted	Nd	Between Lema_G008960 and Lema_G008970
UM317	pKO21	3308800 (11×)	8.8, 8.8	10, 3	342, 42	22	Between Lema_G001330 and Lema_G001340

^a^UM314 had two T-DNA insertion sites; however, only one was identified in this study.

^b^Translocation between Supercontig 5 and Supercontig 17 associated with T-DNA insertion.

*Nd* = Not determined.

In three of the four mutants, the T-DNA was located between genes and in all mutants, the LB and/or RB sequences were partially or completely deleted, possibly explaining why TAIL-PCR failed to identify the T-DNA insertion site. In UM316, no sequences flanking the RB were identified probably due to its proximity to a region of repetitive DNA. In UM176 a large deletion (26 kb) appeared to be associated with a translocation between two Supercontigs (17 and 5), which in the wild-type are on different chromosomes. This rearrangement resulted in deletion of at least 12 genes.

To confirm the location of insertion sites within the genomes of mutant UM176, UM316 and UM317, primers were designed to amplify from inside the T-DNA insert out into the *L. maculans* genome. For all three mutants, PCR amplicons were successfully obtained and sequenced, confirming the location of the insertions (data not shown).

With the decreasing costs of next-generation sequencing, this method of identification of insertion sites in T-DNA mutants is a cost and time-effective solution for both fungi and for plants, where PCR-based methods have not been successful. Furthermore, next-generation sequencing allows identification of chromosomal rearrangements associated with the insertion but not specifically at the insertion site. Such rearrangements are common in both plants [19] and fungi such as the basidiomycete human pathogen, *Cryptococcus neoformans* and the ascomycete rice pathogen *Magnaporthe oryzae* [2],[20]. However, when using Illumina sequencing in situations where the T-DNA has inserted into a highly repetitive region of the genome, as seen in mutant UM316, it might not be possible to identify the precise location of the T-DNA junction and to quantify the amount of fungal DNA deleted during the T-DNA insertion event. Additionally, the presence of multiple T-DNA insertions or repeats within the vector sequences, such as in the case of UM314, can confound the alignment of the border sequences. Therefore all border sequences might not be identified by Illumina sequencing. However, if there is a single T-DNA insertion, in most cases the identification of just one border is sufficient to locate the insertion site and only a small deletion at the insertion site. An advantage of Illumina sequencing, as illustrated in this study, is that as little as 20–30 bp of flanking sequence was sufficient to identify the insertion site of the T-DNA. In contrast, in a previous study, TAIL-PCR of a set of *L. maculans* mutants resulted in 33 flanking sequences of insufficient length to identify the insertion site, and a further 49 sequences gave ambiguous BLAST matches [21].

Similarly, identification of T-DNA insertion sites in mutant lines of the plant *Arabidopsis thaliana* has proven problematic. For example, Ji and Braam (2010) developed restriction site extension PCR, which relies on multiple PCR steps, but this method resulted in recovery of only 21 of 37 flanking sequences [22]. Lepage *et al*. (2013) used Roche 454 genomic sequencing and T-DNA specific primers to identify insertion sites in 55 out of 64 mutants [23]. However, this approach relies on the binding of T-DNA specific primers, which can be problematic if the T-DNA borders are mutated during integration. Furthermore, Roche454 sequencing is relatively expensive. In the current study, Illumina reads of up to 150 bp containing as little as 20–30 bp of *L. maculans* sequence were sufficient for identification of insertion sites; furthermore this approach overcomes potential issues with loss of T-DNA primer binding sites, and chromosomal rearrangements can be identified. With organisms of larger genome size, the amount of sequence data must be proportionately larger to maintain the same likelihood of locating the flanking regions. With the compact size of fungal genomes and the popularity of T-DNA mutagenesis [1]-[6], Illumina sequencing is an ideal method for characterization of insertion sites.
